# Short graft, short tunnel ACL reconstruction with single hamstring and internal brace leads to comparable outcomes to conventional double hamstring technique: A retrospective study

**DOI:** 10.1002/jeo2.70338

**Published:** 2025-07-13

**Authors:** Thana Buranapuntaruk, Thun Itthipanichpong, Chatree Tangpatthanasombat, Danaithep Limskul, Numphung Numkarunarunrote, Sasipa Buranapuntalug

**Affiliations:** ^1^ Department of Orthopedics Chaoprayayomraj Hospital Suphanburi Thailand; ^2^ Department of Orthopaedics, Faculty of Medicine Chulalongkorn University Bangkok Thailand; ^3^ Sports Medicine Research Group, Faculty of Medicine Chulalongkorn University Bangkok Thailand; ^4^ Department of Academic Affairs and Department of Orthopaedics, Faculty of Medicine Chulalongkorn University Bangkok Thailand; ^5^ Department of Radiology, Faculty of Medicine King Chulalongkorn Memorial Hospital Bangkok Thailand; ^6^ Department of Physical Therapy, Faculty of Allied Health Sciences Thammasat University Pathumthani Thailand

**Keywords:** all‐inside technique, anterior cruciate ligament reconstruction, short graft short tunnel ACLR

## Abstract

**Purpose:**

The purpose of our study was to compare (1) ACL graft healing, (2) patient‐reported outcome, and (3) complications after short graft (length < 65 mm), short tunnel (femoral tunnel < 20 mm) single hamstring ACL reconstruction with an internal brace (SGST‐ACLR) technique and double hamstring autograft conventional ACL reconstruction (CON‐ACLR) technique at minimum 2‐year follow‐up.

**Methods:**

A retrospective cohort of patients underwent arthroscopic ACL reconstruction using a hamstring graft, with a minimum 2‐year follow‐up. Graft healing was evaluated at 1 year using a magnetic resonance imaging scan, with the mean signal‐to‐noise quotient ratio (SNQ) measured from three areas: proximal, middle, and distal to the ACL graft. Patients' demographics data, meniscal lesion, chondral lesion, time to operation, time to evaluation, PROMs (International Knee Documentation Committee [IKDC] scores, Tegner activity scale, and Lysholm score), and complications were evaluated. ACL laxity was measured using a side‐to‐side difference (SSD) by a lachmeter.

**Results:**

A total of 51 patients, comprising 25 in the SGST‐ACLR group and 26 in the CONV‐ACLR group, were analysed. The ACL graft diameter was comparable between the two groups (*p* = 0.32). The mean SNQ at 1‐year postoperative MRI showed no significant difference (*p* = 0.21). Furthermore, no statistically significant differences were observed in the postoperative IKDC scores (*p* = 0.36), Lysholm scores (*p* = 0.22), Tegner activity scores (*p* = 0.30), or side‐to‐side differences (*p* = 0.38) at the final follow‐up.

**Conclusion:**

At two years postoperatively, this study demonstrates that SGST‐ACLR with an internal brace provides comparable outcomes in all parameters to CONV‐ACLR. Thus, SGST‐ACLR offers a viable alternative technique for ACL reconstruction, with the added advantage of minimising graft usage.

**Level of Evidence:**

Level IV, retrospective cohort study.

**Abbreviations**
ACL: anterior cruciate ligament
ACLR: anterior cruciate ligament reconstruction
AI‐ACLR: all‐inside ACLR
CON‐ACLR: conventional ACLR
IKDC: International Knee Documentation Committee
MRI: magnetic resonance imaging
PRO: patient‐reported outcome
ROM: range of motion
SNQ: signal‐noise‐quotient
SSD: side‐to‐side difference
STSG‐ACLR: short graft short tunnel ACLR

## INTRODUCTION

The anterior cruciate ligament (ACL) injury is one of the most common sports injuries. The reported ACL graft rupture rate at long‐term follow‐up ranged from 0% to 13.4%. The common cause was technical problems, such as a small graft diameter and uncorrected associated injuries, including ramp lesions, anterolateral ligament injury or posterolateral corner injury [1, 8, 9, 30].

The short graft short tunnel ACL reconstruction (SGST‐ACLR) technique utilises a short graft length to perform ACL reconstruction with a length of less than 6.5 cm [6, 28]. This technique benefits from a larger ACL graft diameter and preserves the gracilis tendon, thereby reducing donor site morbidity and maintaining knee flexion strength [13, 16]. A short tunnel is defined as a tunnel length of less than 20 mm, which may affect the fixation and healing of the ACL graft. However, many studies found no difference in postoperative laxity, functional outcome, and healing [25].

The all‐inside ACL reconstruction technique (AI‐ACLR) creates bone sockets on both the femoral and tibial sides, allowing for minimally invasive reconstruction of the ACL and preservation of the surrounding bone [18, 19]. However, the systematic review and meta‐analysis did not reveal significant differences in patient‐reported outcome scores, laxity, graft maturation, tunnel widening, or rupture rates. One advantage of AI‐ACLR is that it enables ACL reconstruction using a short graft and a secure fixation technique. This technique is beneficial in preserving another hamstring tendon for further operations such as anterolateral ligament reconstruction or posterolateral corner reconstruction, which results in a better functional outcome and reduced donor site morbidity [7, 10, 20, 28].

The SGST‐ACLR technique utilises a short graft tunnel with a single hamstring graft and an internal brace combined with a shortened limb of the ACL TightRope (Arthrex, Naples, FL). This technique ensures an adequate graft diameter while incorporating the same sutured brace without requiring additional material, thereby avoiding extra costs and utilising only a single hamstring tendon [2]. Therefore, this study focused on comparing the functional outcomes, knee stability, MRI‐based graft maturity, and complications of SGST‐ACLR and CONV‐ACLR with a minimum of 2 years of follow‐up postoperatively. We hypothesise that this technique has functional outcomes and complications that are non‐inferior to conventional ACL reconstruction [2].

## MATERIAL AND METHODS

The author retrospectively enroled 51 patients undergoing primary ACLR between 2019 and 2023, which was performed by two surgeons and approved by the hospital's ethics review committee (YM003/2565). The inclusion criteria consist of patients who (1) underwent primary ACLR with hamstring autograft, (2) had adequate follow‐up for 2 years for clinical evaluation, patient‐reported outcomes (PROMs), radiographic evaluation and complications. The exclusion criteria were as follows: (1) revision ACL reconstruction, (2) combined other ligament surgery (e.g., posterolateral ligament reconstruction, medial collateral ligament repair), (3) pre‐existing osteoarthritis with Kellgren and Lawrence (KL) grade ≥ 3 in the knee. Data from patients eligible for this study were collected. Each patient's electrical data was manually reviewed, including demographics, operative records, and postoperative MRI information.

### Surgical technique

**Figure 1:**
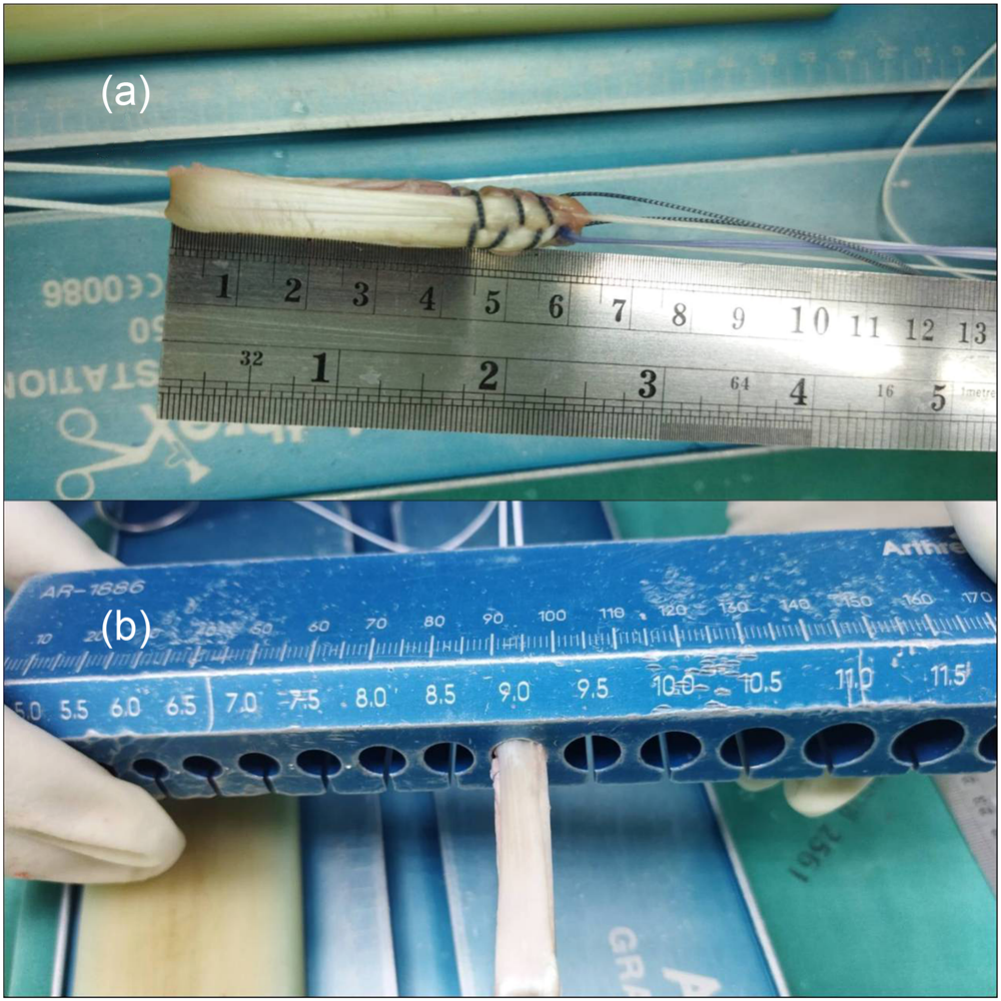
Demonstrate anterior cruciate ligament (ACL) graft preparation. (a) Short ACL graft preparation, approximately 6 cm in length, attached to ACL TightRope (Arthrex, Naples, FL) on both the femoral and tibial sides. (b) The diameter of the ACL graft is approximately 9 mm.

After regional anaesthesia, the patient was placed on the operative table with a side post and footrest. The tourniquet was applied to the limb with a pressure corresponding to the patient's mean arterial blood pressure. An arthroscopic examination confirmed the diagnosis prior to harvesting the hamstring graft. In the SGST‐ACLR group, a semitendinosus tendon was harvested using a tendon stripper and prepared for a quadruple‐loop construct to achieve a diameter of more than 8 mm and a final length of 6.5 cm. If the ACL graft diameter is less than 8 mm, shorten the graft to 6 cm (Figure 1) to increase the diameter. The femoral side was attached to ACL TightRope (Arthrex, Naples, FL), and the tibial side was connected to TightRope (Arthrex, Naples, FL). In the CON‐ACLR group, semitendinosus and gracilis tendon were harvested and prepared for ACL graft with a diameter over 8 mm. and a final length of at least 7.5 mm. The femoral side is attached to ACL TightRope (Arthrex, Naples, FL).

**Figure 2:**
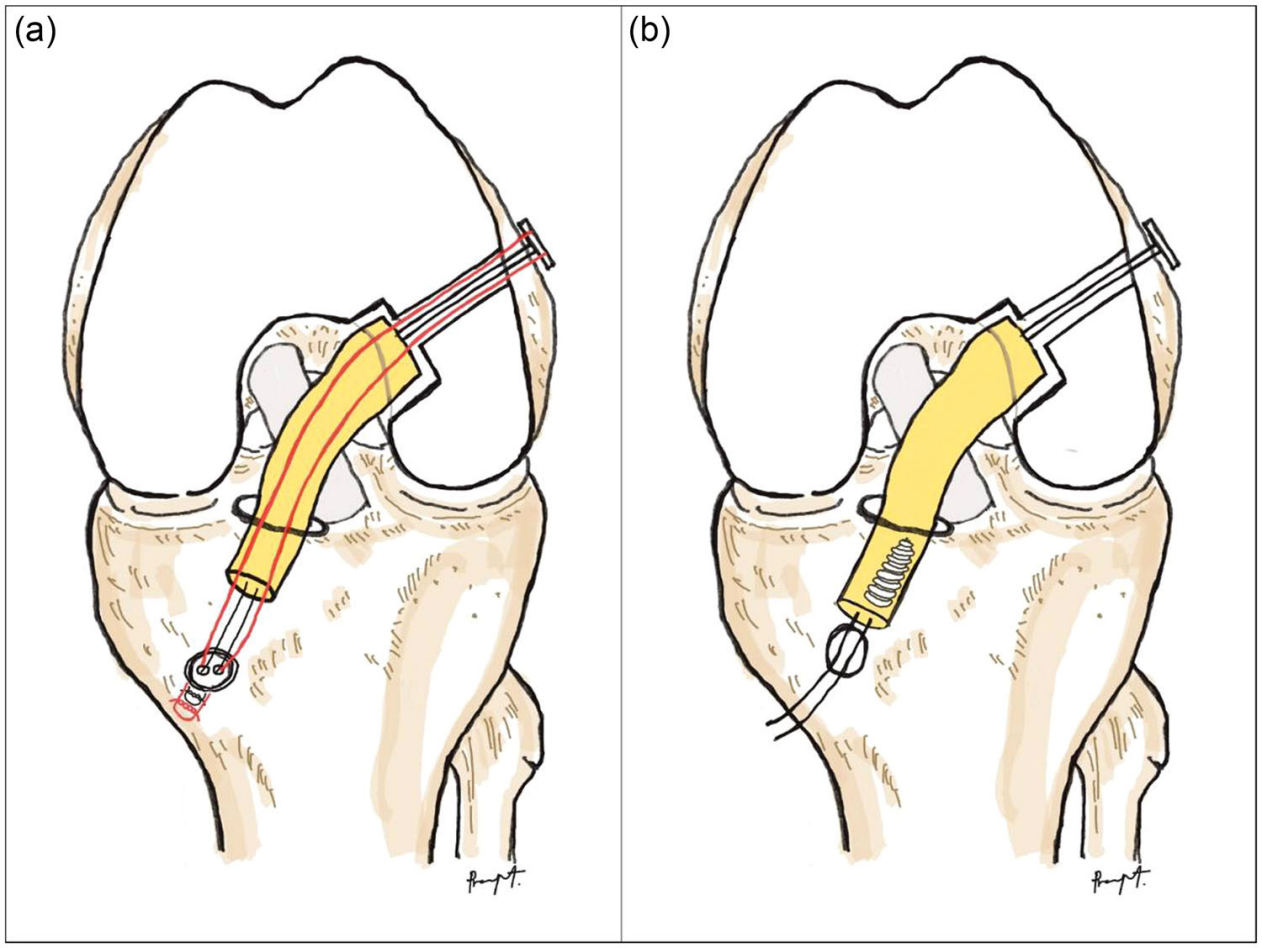
Illustrates ACLR. (a) STSG‐ACLR, with the red line indicating the shortening strand of the femoral adjustable loop passing through the tibial tunnel and tying with the tibial ABS button. (b) CON‐ACLR femoral side fixed ACL TightRope (Arthrex, Naples, FL), tibial side fixed with an interference screw. ACLR, anterior cruciate ligament reconstruction; STSG, short graft short tunnel.

For the SGST‐ACLR group, the femoral socket was reamed with a low‐profile reamer to a depth of approximately 20 mm, and the tibial socket was retrogradely reamed with a FlipCutter (Arthrex) to a depth of approximately 35 mm. The ACL graft was passed through an anteromedial portal to the femoral socket first. Then, the shortening strand was shuttled to engage the graft in the femoral tunnel, approximately 15 mm in length. The ACL graft was then passed through the tibial socket with the shortening strand of the femoral TightRope simultaneously. The cyclic load was applied, and the TightRope ABS (Arthrex) was tensioning in full extension. Then, the shortening strand was re‐tensioned with the ABS button (Arthrex) to adjust the final ACL graft tension to brace the graft. For the CON‐ACLR group, the femoral and tibial sockets were reamed anterogradely with a low‐profile reamer, then fixed the femoral side with ACL TightRope (Arthrex) and Biocomposite interference screw (Arthrex) on the tibial side in the full extension position. Both techniques are illustrated in Figure 2.

The postoperative rehabilitation programme involved initial immobilisation in full extension with a knee brace or slab for 2 weeks. Weight‐bearing depended on the meniscal operation, and range‐of‐motion exercises were performed as soon as the swelling subsided. The isometric quadriceps exercise was performed in the early postoperative period and progressed to bicycling at 4 weeks postoperative. The patents can allow for a return to play after 6–8 months, depending on the specific sport and passing the functional ACL test.

### Outcome measurement

Patients' electronic records were reviewed for demographic data, including sex, age, weight, height, body mass index (BMI), duration of symptoms before surgery and associated injuries, such as meniscal or chondral lesions. Clinical outcomes were measured using patient‐reported outcomes (PROMs), including the International Knee Documentation Committee (IKDC) scores, Tegner activity scale, and Lysholm score [14] at 2 years postoperatively.

**Figure 3:**
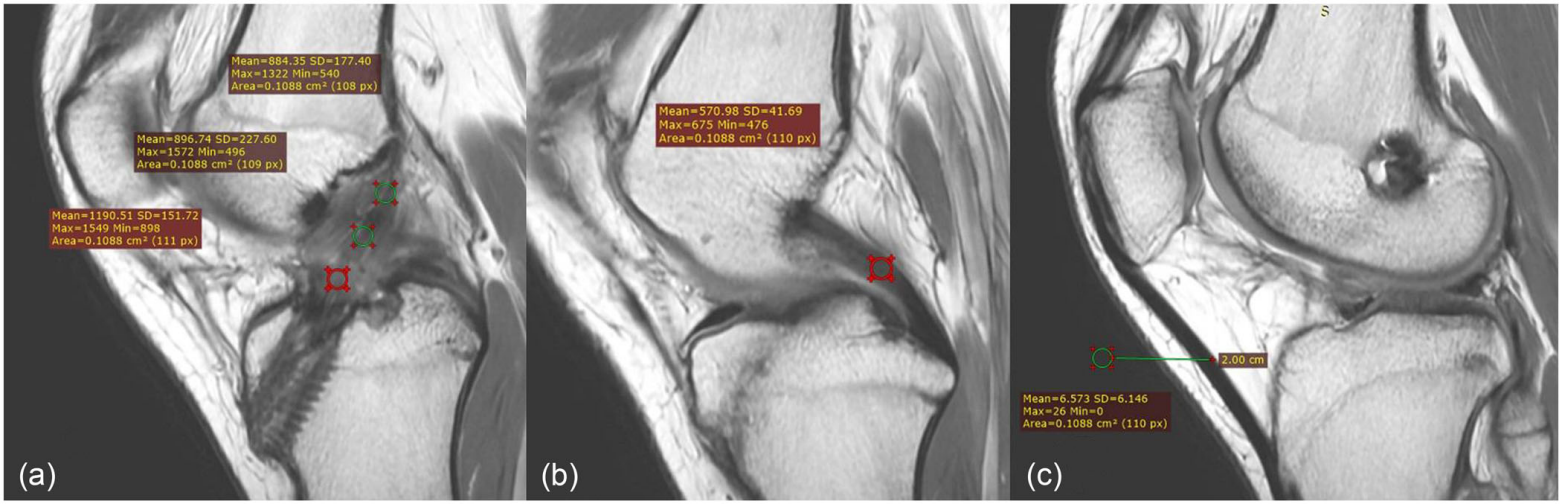
Shown SNQ ratio was measured on the sagittal PD image. (a) Signal intensity was measured at the proximal, middle, and distal ACL graft (b) at the mid‐substance of the PCL and (c) at the background at 2.0 cm anterior to the mid‐point of the patellar tendon. ACL, anterior cruciate ligament; SNQ, signal‐to‐noise quotient.

At 1 year postoperatively, patients were assessed for anterior laxity by measuring the side‐to‐side (SSD) difference with a Lachmeter. The ACL graft was analysed using a fat‐saturated magnetic resonance imaging (MRI) sequence in sagittal planes. The MRI was conducted using a 1.5 Tesla MRI system (Sigma; GE Healthcare), with an imaging thickness of 2 mm. A single musculoskeletal radiologist performed all MRI measurements to ensure consistency, using the RadiAnt DICOM Viewer (Copyright 2009‐2020 Medixant). The signal‐to‐noise quotient (SNQ = graft signal − PCL signal/background signal) was measured by placing a region of interest (ROI) centred at the proximal, middle, and distal regions of the ACL graft in the sagittal view proton density weighted fat suppressed of the same slice [3, 5]. (Figure 3) The femoral and tibial tunnel diameters were measured perpendicular to the long axis of the tunnel at the proximal to distal tunnel and compared with the initial drilled diameter. Even the gold standard is to be measured from a CT scan to limit the cost [26, 27]. The tunnel widening is measured by the maximal diameter of the femoral and tibial tunnel minus the intraoperative diameter of the femur and tibia, respectively.

### Statistics analysis

Statistical analysis was performed using SPSS version 22. Data normality was assessed using the Shapiro–Wilk test. Continuous variables were expressed as mean ± standard deviation (SD) and compared using the unpaired *t*‐test when the data were normally distributed; non‐parametric data were analysed using the Mann–Whitney *U* test. Categorical variables were summarised as counts and percentages and compared using the Chi‐square or Fisher's exact test, as appropriate. Given the number of outcome measures (PROMs, SSD, SNQ, and tunnel widening), adjustments for multiple comparisons were applied using the Bonferroni correction to minimise the risk of type I error; the adjusted significance levels are reported alongside the corresponding *p*‐values. A priori sample size calculations were performed based on previous studies, with an *α* level of 0.05 and a power of 80% (*β* = 0.2), which indicated a minimum of 10 patients per group to detect a clinically meaningful difference in SNQ. Statistical significance was defined as *p* ≤ 0.05 unless otherwise noted after adjustment for multiple comparisons.

## RESULTS

### Patient characteristics

**Figure 4:**
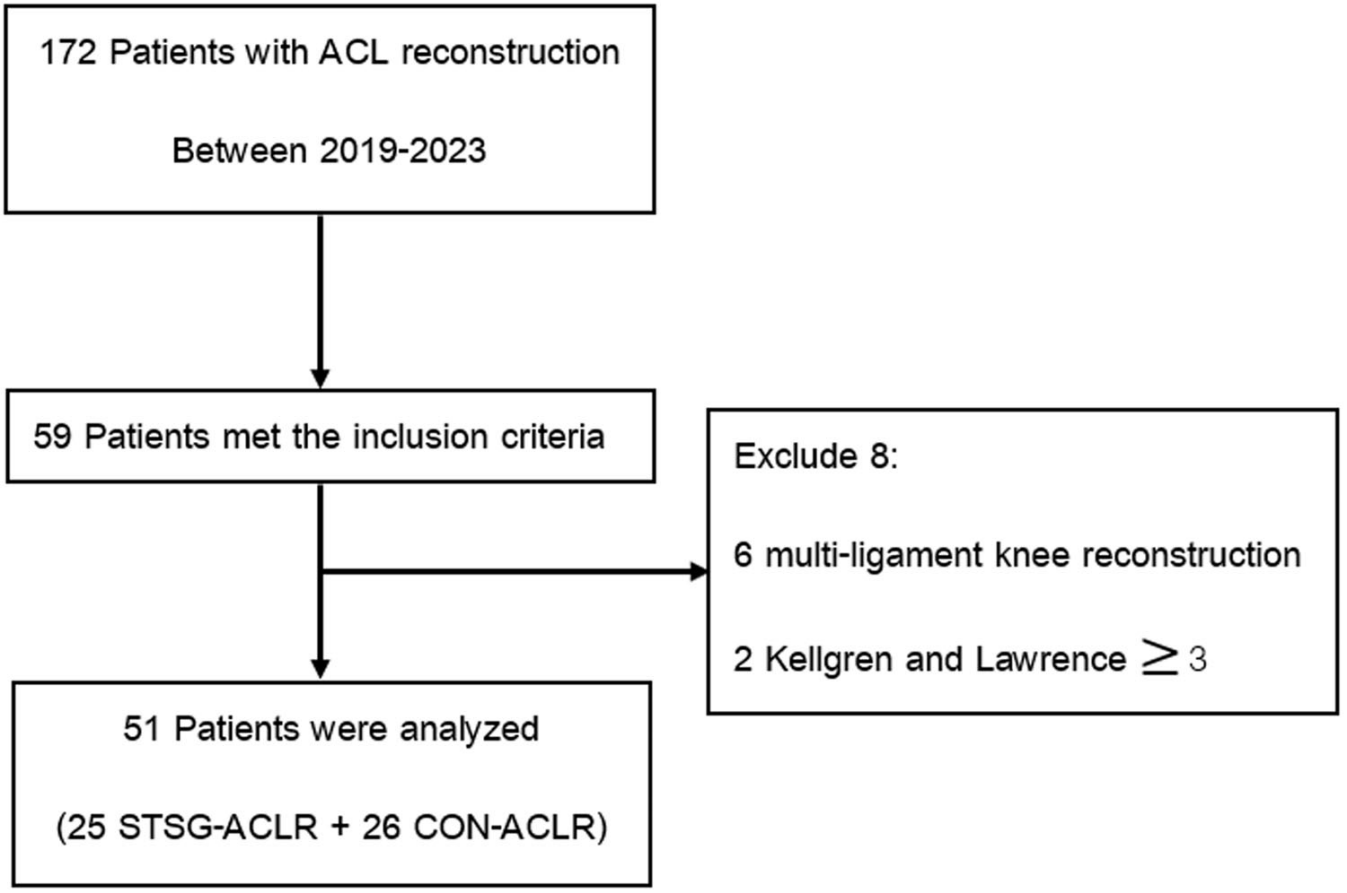
The flowchart illustrates the retrospective analysis of electronic data, focusing on patients who underwent hamstring graft anterior cruciate ligament reconstruction (ACLR). STSG, short graft short tunnel.

One hundred seventy‐two patients who underwent ACL reconstruction between 2019 and 2023 were reviewed. Fifty‐nine patients were identified and met the inclusion criteria. A total of 113 patients were excluded due to loss of follow‐up, ACLR with bone‐patellar tendon, or incomplete postoperative outcomes in any parameter. Six patients were excluded due to multi‐ligament reconstruction, and two were excluded due to osteoarthritis KL grade 3. The final study groups consisted of 51 patients: 25 in the SGST‐ACLR group, with a mean age of 29.8 ± 10.8 years, and 26 in the CON‐ACLR group, with a mean age of 33 ± 10.9 years. (Figure 4) Demographic data are presented in (Table 1), showing no differences in age, sex, BMI and time to reconstruction. The associated meniscal lesion was not different between groups, but there were more chondral lesions than in the CON‐ACLR group. The ACL graft size is equivalent in both groups, with a mean of 8.4 ± 0.5 mm in the SGST‐ACLR and 8.3 ± 0.8 mm in the CON‐ACLR. The mean graft length was 6.48 ± 0.1 cm in the SGST‐ACLR group, shorter than the CON‐ACLR group, which was 7.6 ± 0.4 cm (Table 2).

**Table 1:** Patient characteristics.^a^

Characteristics	SGST‐ACLR (*n* = 25)	CON ACLR (*n* = 26)	*p*‐value
Age (y)	29.8 ± 10.8	33 ± 10.9	0.15
Body mass index	25.2 ± 4.9	25.8 ± 3.8	0.30
Sex			0.11
male	25	22	
female	0	4	
Time to reconstruction, mo. (range)	(1–36)	(1–12)	

Abbreviations: ACLR, anterior cruciate ligament reconstruction; CI, confidence interval; SD, standard deviation; STSG, short graft short tunnel.

^a^ Data are expressed as mean ± SD (95% CI) or *n* (%). The *p*‐value indicates a statistically significant difference between groups (*p* < 0.05).

**Table 2:** Patient injury characteristics, ACL graft size, and additional procedures.^a^

Characteristics	SGST‐ACLR (*n* = 25)	CON ACLR (*n* = 26)	*p*‐value
Meniscal injury	20 (80)	19 (73)	>0.99
Meniscal repair or partial meniscectomy	20 (80)	19 (73)	>0.99
Chondral lesion	2 (8)	7 (26)	>0.99
ACL graft size (mm)	8.4 ± 0.5	8.3 ± 0.8	0.32
ACL graft length (cm)	6.48 ± 0.1	7.6 ± 0.4	>0.99
Femoral tunnel (mm)	8.3 ± 0.5	8.2 ± 0.8	0.37
Tibial tunnel (mm)	8.6 ± 0.5	8.9 ± 0.8	0.33

Abbreviations: ACLR, anterior cruciate ligament reconstruction; CI, confidence interval; SD, standard deviation; STSG, short graft short tunnel.

^a^ Data are expressed as mean ± SD (95% CI) or *n* (%). The *p*‐value indicates a statistically significant difference between groups (*p* < 0.05).

### Clinical outcomes

**Table 3:** Patient clinical outcomes at final follow‐up.^a^

Outcome	SGST‐ACLR (*n* = 25)	CON ACLR (*n* = 26)	*p*‐value
IKDC score	79.3 ± 8.3	80.1 ± 9.5	0.36
Lysholm score	94.5 ± 4.4	95.5 ± 4.6	0.22
Tegner score	7.6 ± 1.3	7.4 ± 1.4	0.3
Side‐to‐side difference, mm	1.6 ± 1.1	1.7 ± 1.5	0.207
Complication	0	2	0.49
Graft failure	0	1	1.0
Infection	0	1	1.0

Abbreviations: ACLR, anterior cruciate ligament reconstruction; CI, confidence interval; IKDC, International Knee Documentation Committee; SD, standard deviation; STSG, short graft short tunnel.

^a^ Data are expressed as mean ± SD (95% CI) or *n* (%). The *p*‐value indicates a statistically significant difference between groups (*p* < 0.05).

The postoperative PROMs were not different between the two groups (Table 3). The mean postoperative IKDC score (SGST‐ACLR vs. CON‐ACLR: 79.3 ± 8.3 vs. 80.1 ± 9.5; *p* = 0.36), Lysholm (SGST‐ACLR vs. CON‐ACLR: 94.5 ± 4.4 vs. 95.1 ± 4.6; *p* = 0.22), and Tegner activity score (SGST‐ACLR vs. CON ACLR: 7.6 ± 1.3 vs. 7.4 ± 1.4; *p* = 0.3) shown no significance distinction. The mean side‐to‐side difference was 1.6 ± 1.1 mm in the SGST‐ACLR group and 1.7 ± 1.5 mm in the CON‐ACLR group, with no statistically significant difference (*p* = 0.20).

### Radiographic outcome

**Table 4:** Patient radiographic outcomes at final follow‐up.^a^

Outcome	SGST‐ACLR (*n* = 25)	CON ACLR (*n* = 26)	*p*‐value
SNQ ratio mean	78.9 ± 96.3	61.6 ± 57.7	0.21
SNQ proximal	85.7 ± 104	51.2 ± 50.3	0.06
SNQ middle	87.2 ± 93.7	65.1 ± 63.1	0.16
SNQ distal	67.8 ± 94.8	67.7 ± 65.7	0.49
Femoral tunnel widening (mm)	1.7 ± 0.7	1.6 ± 0.9	0.05
Tibial tunnel widening (mm)	2.2 ± 1.2	2.4 ± 1.4	**0.008**

Abbreviations: ACLR, anterior cruciate ligament reconstruction; CI, confidence interval; IKDC, International Knee Documentation Committee; SD, standard deviation; SNQ, signal‐to‐noise quotient; STSG, short graft short tunnel.

^a^ Data are expressed as mean ± SD (95% CI) or *n* (%). The *p*‐value indicates a statistically significant difference between groups (*p* < 0.05).

At the 1‐year follow‐up, the mean SNQ was 78.9 ± 96.3 (proximal 85.7 ± 104, middle 87.2 ± 93.7 and distal 67.8 ± 94.8) in the SGST‐ACLR group, and the mean SNQ was 61.6 ± 57.7 (proximal 51.2 ± 50.3, middle 65.1 ± 63.1 and distal 67.7 ± 65.7) in the CON‐ACLR group (*p* = 0.21) (Table 4). The mean femoral tunnel widening in the SGST‐ACLR group was 1.7 ± 0.7 mm (range, 1–3 mm), while in the CON‐ACLR group, it was 1.6 ± 0.9 mm (range, 0–4.5 mm) (*p* = 0.05). The mean tibial tunnel widening in the SGST‐ACLR group was 2.2 ± 1.2 mm (range, 0–3 mm), whereas in the CON‐ACLR group, it was 2.4 ± 1.4 mm (range, 0–5.5 mm) (*p* = 0.008). There were no statistically significant differences between groups in any of the parameters.

### Complication

There was one graft rupture after return to sport in the CON‐ACLR group and a revision ACLR with bone‐patellar‐bone graft 1 month after the accident (Table 3). There was one postoperative infection in the CON‐ACLR group, which was successfully treated with early arthroscopic debridement and graft preservation.

## DISCUSSION

This study introduced the short graft, short tunnel all‐inside ACL reconstruction technique with a recycling internal brace to increase the ACL graft diameter and preserve the gracilis tendon. The results of PROMs did not differ between groups at the final follow‐up, supporting the notion that SGST‐ACLR yields the same results as CON‐ACLR. The results demonstrate that the SGST‐ACLR technique has no difference in graft diameter compared to the CON‐ACLR technique, with the same PROMs, side‐to‐side difference, and signal on MRI. The SSD was also similar, suggesting that the SGST‐ACLR did not compromise graft stability. There was a significant difference in tibial tunnel widening, from 2.2 ± 1.2 mm in the SGST‐ACLR group to 2.4 ± 1.4 mm in the CONV‐ACLR group, but this difference did not have clinical significance. However, at a 2‐year follow‐up, the graft failure rates of both groups do not differ.

Both groups exhibited high SNQ with a wide standard deviation, which may be attributed to imaging protocols, such as proton density and T2‐weighted fat suppression, that enhance ligament contrast, resulting in a high SNQ ratio. Additional shortening strands of ACL TightRope (Arthrex, Naples, FL) made from fibre wire to ACL graft can cause high SNQ due to partial volume effects. However, the wide standard deviation may be attributed to biological variability in graft healing, revascularization, and remodelling among patients. These factors highlight the complexity of MRI signal interpretation in ACLR and suggest the need for a standardised imaging protocol to minimise variability in the future [29, 31].

Numerous studies have been conducted to determine the minimum tunnel length that promotes the healing of ACL grafts. The femoral socket should generally be at least 20 mm in diameter due to concerns regarding graft healing and biomechanics. Cavaignac et al. demonstrated no difference in bone incorporation and ligamentization on MRI at 1 year postoperatively between femoral tunnel lengths of 10 and 20 mm [3]. Moon et al. compared three groups of femoral graft insertional lengths with tibialis anterior allografts: less than 15 mm, 15–20 mm, and more than 20 mm. They show no significant difference in the postoperative functional outcome and knee laxity [22]. Gupta et al. showed no difference in clinical and radiological outcomes between grafts in the femoral tunnels with a diameter of more than 20 mm and less than 20 mm.

Data from numerous systematic reviews and meta‐analyses indicate that the AI‐ACLR technique yields favourable clinical outcomes and joint stability comparable to those of the conventional method [12, 17, 20]. The advantage of AI‐ACLR is bone preservation, enabling the retention of the ACL graft, and giving strong construction, especially when fixation STSG‐ACLR that cannot be secure with an interference screw. However, concerns exist regarding the potential for loop lengthening of the adjustable suspensory device [23, 24].

Our STSG‐ACLR, which features a recycling internal brace, utilises a shortening limb of femoral suspensory fixation, similar to an internal brace, tied with tibial suspensory fixation to prevent loop lengthening [2]. This technique has the advantage of preserving the gracilis tendon for better knee flexion strength [16, 21]. The gracilis tendon can be preserved for future use in operations such as anterolateral ligament reconstruction or posterolateral corner reconstruction. The strength of the ACL graft is the same as that of the CON‐ACLR technique because a short ACL graft can be prepared in quadruple, making the ACL graft larger with a recycling internal brace to prevent the graft and fixation from lengthening [14, 15]. Our study's results revealed that postoperative stability and ligamentization, as assessed by MRI, are comparable to those of the CON‐ACLR technique.

A low ACL graft diameter is associated with an increased risk of ACL graft rupture. The graft diameter should be more than 8 mm to reduce the re‐rupture rate [11]. However, in the Asian population, the hamstring graft is often too short and small, particularly in female adults [4]. The SGST‐ACLR technique offers the benefit of a larger graft diameter with the same graft tissue, while sparing the gracilis tendon to reduce donor site morbidity, pain, and improve knee flexion strength. The all‐inside technique is used to secure short grafts, providing good stability.

## LIMITATION

This study has certain limitations. It is retrospective and includes a relatively small number of cases, with no preoperative comparison of patient‐reported outcomes (PROMs) between the groups. Only one musculoskeletal radiologist performed all measurements, which improved consistency, but there were no inter‐ or intra‐observer reliability statistics. Additionally, the SGST‐ACLR technique incorporates a recycled internal brace to enhance the strength of the graft. As a result, it remains unclear whether the single hamstring all‐inside technique, without the internal brace, achieves outcomes comparable to those of the double hamstring approach.

## CONCLUSION

The SGST‐ACLR demonstrates outcomes comparable to those of conventional ACLR in terms of functional performance, laxity, ligamentization, femoral tunnel widening, and rupture rates at a 2‐year follow‐up. While MRI findings reveal significant tibial tunnel widening in the SGST‐ACLR group, this has no clinical relevance. Notably, the SGST‐ACLR offers the distinct advantage of requiring only a single hamstring tendon, making it a resource‐efficient for ACL reconstruction.

## AUTHOR CONTRIBUTIONS

Design, manuscript writing, and data curation: Thana Buranapuntaruk. Responsible for revising the article: Thun Itthipanichpong. Data analysis and interpretation: Danaithep Limskul. Radiographic measurement: Numphung Numkarunarunrote. Data collection and assembly: Chatree Tangpatthanasombat. Statistical analysis and editing of the article: Sasipa Buranapuntalug.

## CONFLICT OF INTEREST STATEMENT

The authors declare no conflicts of interest.

## ETHICS STATEMENT

This study was approved by the Institutional Review Board of Hospital (YM003/2565) and TCTR20220629005.

## Data Availability

The data are available from the corresponding author on reasonable request.
